# Facile synthesis of silicon nitride nanowires with flexible mechanical properties and with diameters controlled by flow rate

**DOI:** 10.1038/srep45538

**Published:** 2017-03-28

**Authors:** Shun Dong, Ping Hu, Xinghong Zhang, Yuan Cheng, Cheng Fang, Jianguo Xu, Guiqing Chen

**Affiliations:** 1 Science and Technology on Advanced Composites in Special Environment Laboratory, Harbin Institute of Technology, Harbin 150001, PR China

## Abstract

Ultralong Si_3_N_4_ nanowires (NWs) were successfully synthesized with size controlled in N_2_ gas by using an efficient method. The diameters of the Si_3_N_4_ NWs increased when the flow rate of N_2_ gas increased, with average diameters of 290 nm from flow rates of 100 ml/min, 343 nm from flow rates of 200 ml/min and 425 nm from flow rates of 400 ml/min. Young’s modulus was found to rely strongly on the diameters of the Si_3_N_4_ NWs, decreasing from approximately 526.0 GPa to 321.9 GPa; as the diameters increased from 360 nm to 960 nm. These findings provide a promising method for tailoring these mechanical properties of the NWs in a controlled manner over a wide range of Young’s modulus values. Vapour-liquid-solid (VLS) mechanisms were used to model the growth of Si_3_N_4_ NWs on the inner wall of an alumina crucible and on the surface of the powder mixture. Alumina may be an effective mediator of NW growth that plays an important role in controlling the concentrations of Si-containing reactants to support the growth of NWs on the inner wall of the alumina crucible. This approach offers a valuable means for preparing ultralong Si_3_N_4_ NWs doped with Al with unique properties.

Since the discovery of carbon nanotubes, one-dimensional (1D) nanomaterials have attracted wide attention due to their unique microstructures and properties and their great potential applications^1^,^2^,^3^,^4^,^5^. As one of most common 1D nanomaterials, 1D Si_3_N_4_ nanomaterials (e.g., NWs and nanobelts (NBs)) not only possess the outstanding properties of their bulk counterparts, such as thermomechanical properties and chemical inertness^6^,^7^, but also have their own unique properties, including superior photoelectric and mechanical properties, due to the quantum confinement effect^8^,^9^,^10^, which could facilitate their wide used in nanocomposites and nanoelectronics^11^,^12^. Moreover, several techniques have been applied to prepare 1D Si_3_N_4_ nanomaterials, including the pyrolysis of polymeric precursors, carbothermal reduction, and chemical vapor deposition (CVD)^13^,^14^,^15^. Meanwhile, Si_3_N_4_ nanomaterials with lengths on the order of millimeters or even centimeters could be more valuable in some fields (e.g., connections for devices and reinforcements for composites) compared with shorter versions^16^,^17^,^18^. Thus, great efforts have been made to prepare ultralong Si_3_N_4_ nanomaterials with high yields. For example, Gao *et al*. synthesized ultralong, single-crystalline α-Si_3_N_4_ NBs by pyrolysis of a polymeric precursor without any template, in which pyrolysis was performed at 1550 °C in a conventional tube furnace under an atmosphere of ultrahigh purity nitrogen^19^. Lin *et al*. successfully obtained ultralong Si_3_N_4_ nanomaterials as well by a CVD route under superatmospheric pressure conditions, in which CH_4_ was used as the reducing gas^20^. Although ultralong Si_3_N_4_ nanomaterials could be produced by the above methods, these approaches involved either complex processes or severe demands on the equipment (e.g., superatmospheric pressures). Therefore, it is still necessary to develop simple and effective methods to prepare ultralong Si_3_N_4_ nanomaterials.

Accurate measurements of the mechanical behaviour and properties of 1D Si_3_N_4_ nanomaterials are very important for their integration into functional nanodevices, which case mechanical failure would lead to the malfunction or failure of the nanodevice^21^. Various approaches for measuring the mechanical behaviour and properties of individual nanomaterials have been employed up to now, such as nanoindentation, *in situ* bending and tensile tests^22^. For instance, Saini *et al*. mechanically designed helically coiled carbon NWs and studied their resonance modes using the harmonic detection of resonance technique, which showed that the shear moduli of the NWs were approximately 8 ± 2 GPa^23^. Ma *et al*. measured the young’s moduli of SiO_2_@SiC NWs by using the three-point-bending test, which showed that the influence of the oxide sheath on the modulus should not be ignored^24^. Although there are a significant number of reports in the literatures on tests of the mechanical properties of NWs, the mechanical behaviour and properties of Si_3_N_4_ NWs have rarely been reported according to our survey.

Therefore, the objective of this paper is to develop a simple and effective method to synthesize ultralong Si_3_N_4_ NWs with size controlled and to characterize the mechanical properties of individual NWs by using a hybrid SEM/SPM system with *in situ* nanoindentation, which is a powerful method for manipulating and characterizing the properties of individual nanostructures^24^. To the best of our knowledge, this is the first time that the mechanical properties of Si_3_N_4_ NWs have been measured with a hybrid SEM/SPM system by using *in situ* nanoindentation.

## Results and Discussion

### Characterization of Si_3_N_4_ NWs

**Figure 1:**
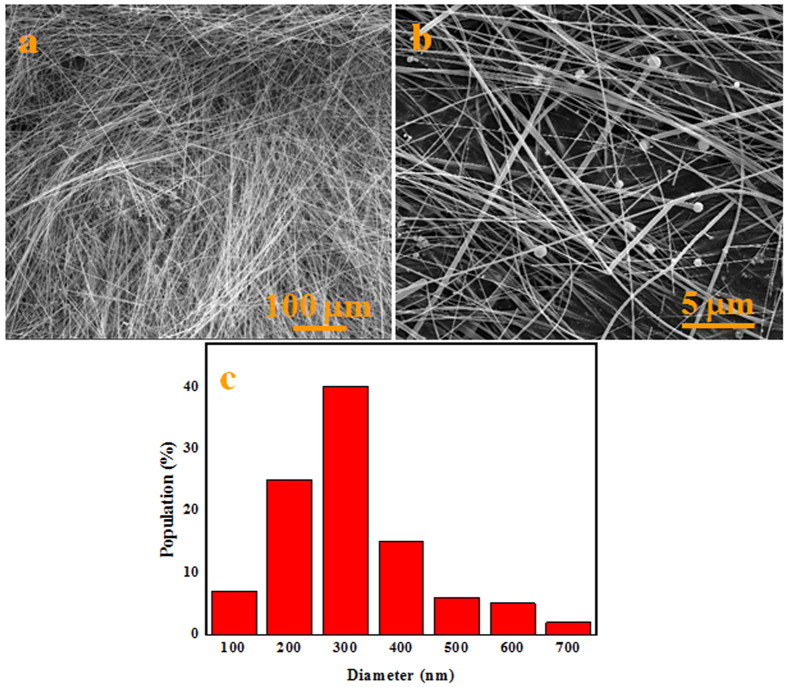
SEM images and the width distribution histogram of the white wool-like products obtained on the inner wall of ceramic crucible.

**Figure 2:**
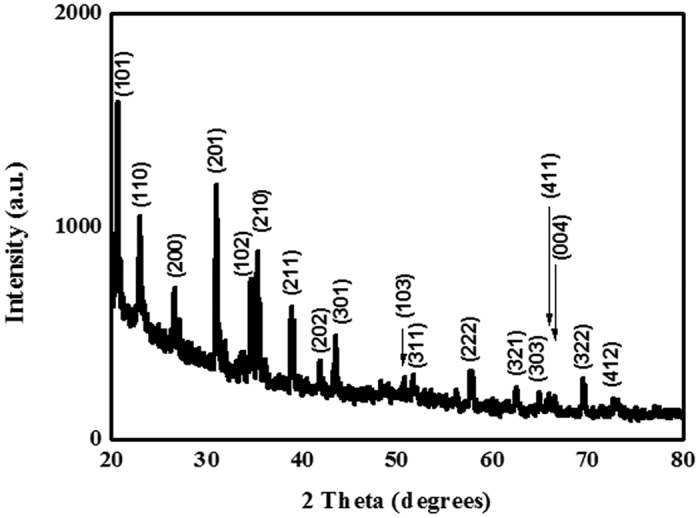
XRD pattern of white wool-like products obtained on the inner wall of ceramic crucible.

**Figure 3:**
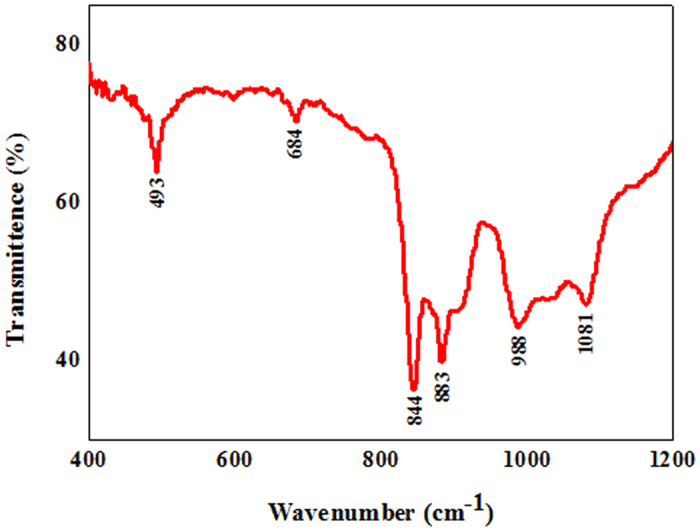
FTIR spectrum of white wool-like products obtained on the inner wall of ceramic crucible.

**Figure 4:**
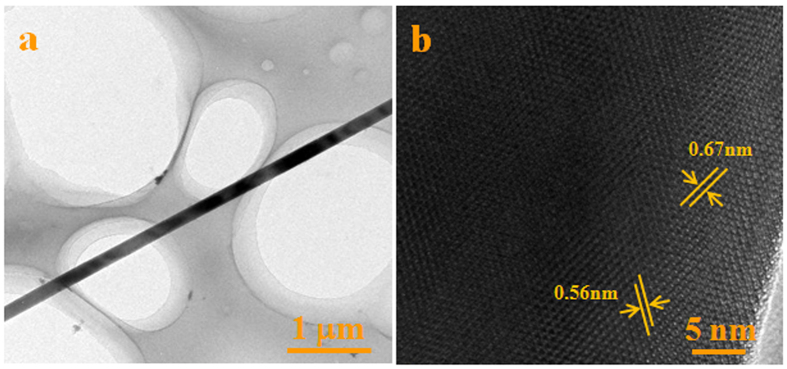
TEM and HRTEM images of a single Si_3_N_4_ NW obtained on the inner wall of ceramic crucible.

The morphology and microstructures of the synthesized white wool-like products were obtained on the inner wall of the alumina crucible at a flow rate of 100 ml/min N_2_ and were examined by SEM, as shown in Fig. 1. The products exhibited wire-like with lengths of several hundred micrometres in the low-magnification SEM image (Fig. 1a), while the lengths of the white wool-like products obtained on the inner wall of the alumina crucible and on the surface of mixed powder were up to several millimeters, as determined from the macroscopic morphology observed using a digital camera and as shown in Fig. S1. A diameter distribution histogram of the NWs (Fig. 1c) from the statistical measurements based on SEM images showed an average diameter of 290 nm and a distribution ranging 200–400 nm. Meanwhile, some droplets were observed at the tips of the NWs, and the composition of these droplet were also analysed by EDS equipped with SEM (the results are shown in Fig. S2). It was observed that these droplets were composed of Si, N, C, Fe and O (Au came from the preparation of the samples for improving electrical conductivity), which suggests that the growth of the NWs could be governed by a typical VLS growth mechanism and that Fe could be the catalyst to support the growth of the NWs^25^,^26^,^27^. The white wool-like products formed on the inner wall of the alumina crucible were ground to powder to confirm their crystalline phase. A typical XRD pattern, as shown in Fig. 2, revealed three major peaks in terms of intensity that were assigned to the (101), (110) and (201) planes, which were characteristic of α-Si_3_N_4_ and suggested that the main composition of the white wool-like products was α-Si_3_N_4_^28^,^29^. To further confirm the composition of the white wool-like products, FTIR measurements were performed, and a typical FTIR transmittance spectrum is shown in Fig. 3. A broad band was observed in the range of 800–1100 cm^−1^, with two strong absorption peaks at approximately 844 and 883 cm^−1^ that can be attributed to the Si-N stretching vibration mode of α-Si_3_N_4_ based on previous literature^30^,^31^. In addition, the absorption peaks at 1081 cm^−1^ and 988 cm^−1^ showed a blueshift compared with the peaks of bulk α-Si_3_N_4_, which was likely due to size- and/or surface-induced quantum effects^6^,^32^. Moreover, the peak located at 493 cm^−l^ was assigned to the stretching motion of N-Si bonds, and the peak at approximately 684 cm^−1^ was ascribed to a Si-Si stretching mode^6^,^14^,^32^. Therefore, it was reasonable to believe that the white wool-like products were pure α-Si_3_N_4_. The nanostructure of Si_3_N_4_ NWs was further characterized by TEM and HRTEM as shown in Fig. 4. The illustrative TEM image (shown in Fig. 4a) revealed that the α-Si_3_N_4_ NWs were uniform in diameter along their entire length and had perfect crystalline structures with few defects. A typical HRTEM image of a single Si_3_N_4_ NW is shown in Fig. 4b, and the inset lattice-fringe spacing of 0.67 nm and 0.56 nm agreed well with the (001) and (100) planes of α-Si_3_N_4_, respectively^6^,^14^. Moreover, the extension direction of the α-Si_3_N_4_ NWs should coincide with the [101] crystal axis, suggesting that the crystal growth direction of the NWs might be along the [101] direction^14^. In summary, this method supplies a promising means for preparing ultralong Si_3_N_4_ NWs with few defects on an industrial scale.

### Synthesis of Si_3_N_4_ NWs with controlled size

**Figure 5:**
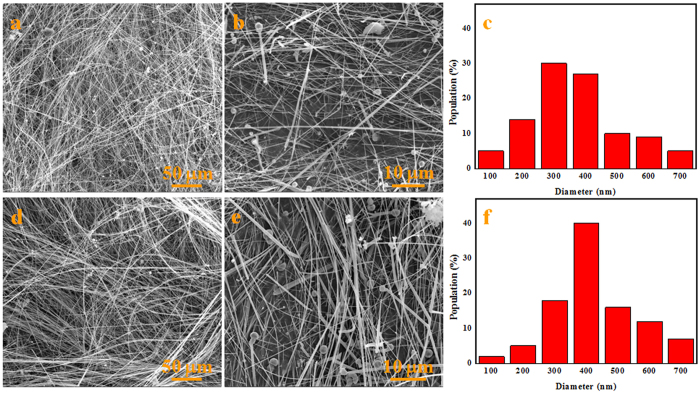
SEM images and the width distribution histograms of the products obtained in different flow rates of N_2_ at 1400 °C. The flow rates were (**a**–**c**) 200 ml/min and (**d**–**f**) 400 ml/min.

To date, many efforts have been made to fabricate NWs with desired properties. In this study, the influence of N_2_ flow rate on the size of Si_3_N_4_ NWs was investigated^33^,^34^,^35^. The macroscopic morphology of the products obtained from 200 ml/min and 400 ml/min N_2_ flow rates are shown in Fig. S3. The yields and lengths of these products obviously decreased when the flow rate of N_2_ increased. When the flow rate of N_2_ was 200 ml/min, some several millimeters-long NWs were observed on the inner wall of the alumina crucible and on the surface of the mixed powder were observed, while only a few several millimeters-long NWs on the inner wall of the alumina crucible and no obvious white wool-like products were found on the surface of the mixed powder. In addition, the microstructures of the products were analysed by SEM, as shown in Fig. 5 along with the diameter distribution histograms of the NWs. From the low-magnification SEM images (Fig. 5a and d), it was observed that the morphologies of the NWs were similar to the morphologies of the NWs obtained at different flow rates, with both straight and curved shapes. Meanwhile, some droplets located at the tips of NWs prepared with different flow rates were observed in the high-magnification SEM images (Fig. 5b and e). Notably, the diameters of the NWs obtained at different flow rate were different, as shown in Fig. 5c and f, with the diameter distribution histogram revealing that (i) the NWs grown at 200 ml/min had an average diameter of 343 nm with a main distribution ranging from 300 nm to 400 nm, and (ii) the NWs grown at 400 ml/min had an average diameter of 425 nm with a main distribution ranging from 300 nm to 500 nm. Therefore, it was reasonable to believe that the 100 ml/min flow rate of N_2_ was more suitable for preparing Si_3_N_4_ NWs than the higher N_2_ flow rates. This approach provides an effective method for fabricating Si_3_N_4_ NWs with the size controlled by changing the flow rate of N_2_.

Although the growth mechanisms are not yet clearly understood, the flow rate of nitrogen gas played a pivotal role in controlling the diameters of the Si_3_N_4_ NWs, and an explanation is presented here for discussion. Based on the droplets located at the tips of NWs, the VLS mechanism is the most suitable for explaining the growth of NWs with a tip growth model, because one characteristic of the tip growth model is that the size and shape of the alloying droplets determine the size and shape of the NWs grown^36^. Increasing of flow rate of N_2_ could lead to a higher concentration of N_2_, enhancing the degree of supersaturation of the gaseous reactants and the driving force of the chemical reaction, similar to the effect of increasing the CH_3_SiCl_3_ content on the growth of SiC NWs observed in previous literature^37^. The high degree of supersaturation of the gaseous reactants might result in changes of the size and shape of the alloying droplets, resulting in the formation of large diameter Si_3_N_4_ NWs, while the higher reaction energy could also promote Si_3_N_4_ NWs growth along the radial direction^37^,^38^. Meanwhile, increasing of the flow rate of N_2_ would restrict the axial growth, which could be relevant to the insufficient time between the gaseous reactants for the reaction. This conclusion could be employed to explain why the lengths of the NWs decreasing resulted from increasing the flow rate of N_2_^37^,^38^. In addition, the temperature of 1400 °C could provide enough energy for the nucleation barrier to occur in the formation of the Si_3_N_4_ NWs as the flow rate of N_2_ changed from 100 ml/min to 400 ml/min^37^,^38^.

### Growth mechanism of Si_3_N_4_ NWs obtained at different locations

Based on the macroscopic morphologies of products shown in Figs S1 and S3, it was observed that the yields and lengths of the products growth on the inner wall of the alumina crucible were larger than those of the products grown on the surface of the powder mixture. This phenomenon could be attributed to the different growth models of products obtained at different locations, which is analysed in detail in the following section.

**Figure 6:**
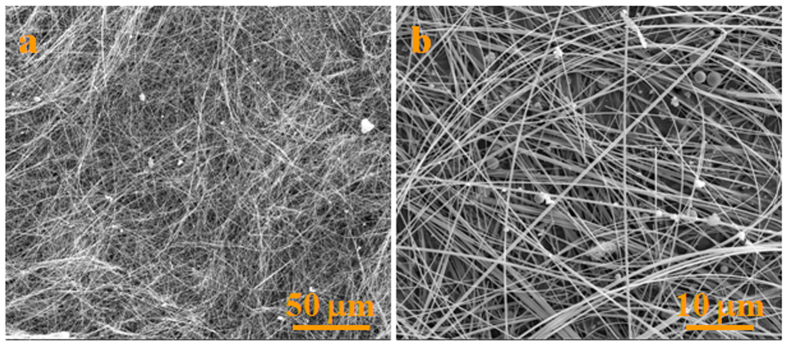
SEM images of the white wool-like products obtained on the surface of the powder mixture.

First, the morphology of the white wool-like products on the surface of the powder mixture was examined by SEM, and typical SEM images are shown in Fig. 6. The NWs also exhibited straight and curved shapes, similar to those of the NWs grown on the inner wall of the alumina crucible. There were also some droplets located at the tips of the NWs, suggesting that the growth of these NWs could also be explained by a typical VLS mechanism. Moreover, the composition of droplet located at the tip of a NW from the surface of the powder mixture was analysed by EDS, as shown in Fig. S4, which indicated that the droplet consisted of Si, N, C and Fe. Fe most likely came from a small amount of Fe_2_O_3_ impurity in the raw material of SiC powder, since no catalyst was employed in the present experiment. A comparison experiment with pretreated powders was carried out to confirm this theory, in which the SiC was first treated in HCl for 60 h to remove Fe completely and then was mixed with 10 wt% polycarbosilane (PCS), while the other experimental parameters were kept constant. The macroscopic morphology of products of this experiment is revealed in Fig. S5. It was observed that no obvious white wool-like products were grown on the surface of mixed powder, demonstrating that the Fe from the SiC powder played a vital role in the growth of the *in*-*situ* NWs. Owing to the presence of Fe in the powder, Fe_2_O_3_ would be reduced to active Fe nanoclusters under the reductive gas from the pyrolysis of PCS (e.g., CO and H_2_) in the experiment. Meanwhile, gaseous silane fragments, hydrocarbon fragments and H_2_ would be released at high temperature from the pyrolysis of PCS^39^. Furthermore, active Fe nanoclusters would become preferable sites to absorb the gaseous silane fragments containing Si, C elements and N_2_, and those gases would be further decomposed and dissolved into the Fe nanoclusters^40^. The absorbed gaseous containing Si, C and N elements would dissolve into the molten droplets to form a supersaturated solution, and then form the Si_3_N_4_ phase, i.e., the Si_3_N_4_ nuclei. Furthermore, Si_3_N_4_ NWs would subsequently grow along a fixed direction as long as the reactant gases needed for the growth of Si_3_N_4_ NWs were continuously generated by the pyrolysis of PCS^40^. Therefore, the NWs growth on the surface of the powder mixture was ruled by the VLS mechanism.

**Figure 7:**
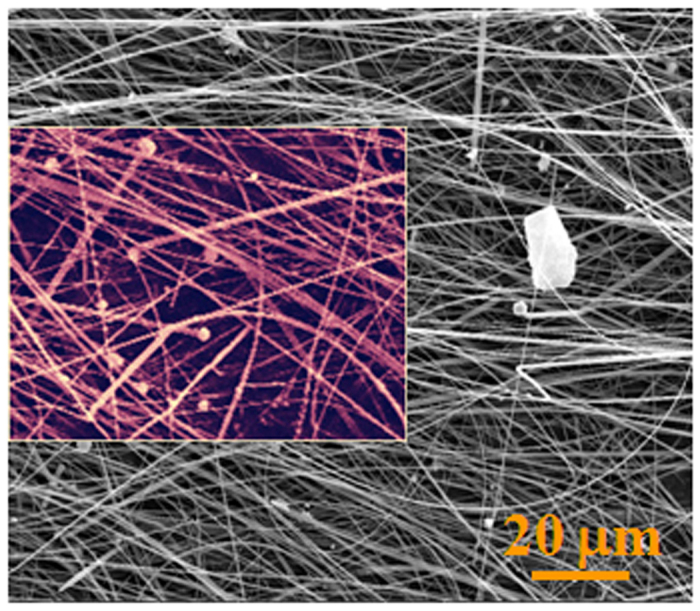
SEM image of the white wool-like products marked in A by a yellow quadrilateral in Fig. S5.

**Figure 8:**
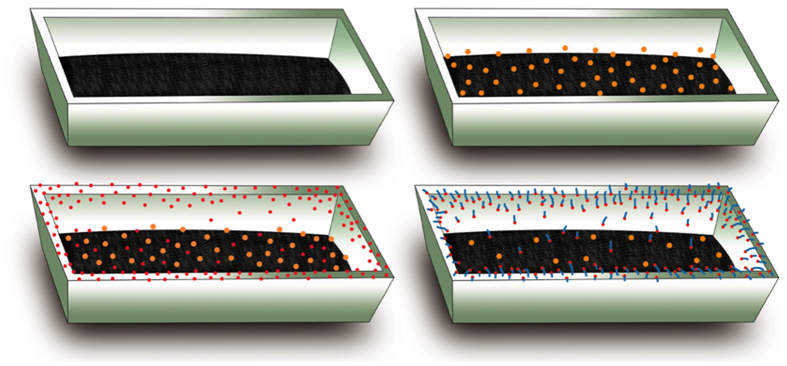
A schematic illustration for the growth process of Si_3_N_4_ NWs.

**Figure 9:**
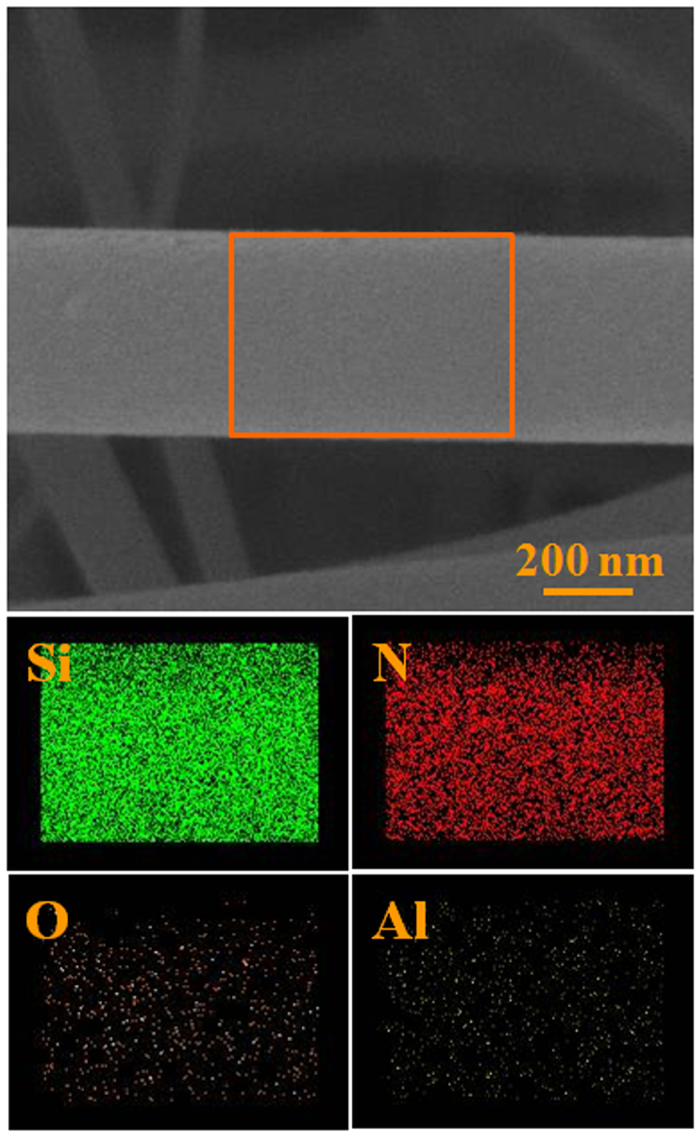
Elemental area scanning of a single Si_3_N_4_ NW.

Thanks to the presence of Fe in the droplet located at the tip of the NW, as shown in Fig. S2, the growth model for Si_3_N_4_ NWs grown on the inner wall of the alumina crucible was determined to be similar to the Si_3_N_4_ NWs grown on the surface of the powder mixture. The source of the Fe might be from the evaporation of active Fe nanoclusters formed in the powder mixture. It is worth noting that only a few white wool-like products were grown on the inner wall of the alumina crucible, while no obvious white wool-like products were obtained on the surface of the powder mixture, suggesting that the Fe in the powder mixture was removed completely, as shown in Fig. S5. The microstructures of white wool-like products grown from the inner wall of the alumina crucible is marked in A by a yellow quadrilateral in Fig. 7, while a few of droplets were located at the tips of the NWs, with the composition of droplet also containing Fe, similar to the result shown in Fig. S2, suggesting that the Fe also came from other source. In addition to other possible sources, Fe might come from the crucible, which contains alumina and a small amount of Fe_2_O_3_ and SiO_2_ impurities. Fe_2_O_3_ from the crucible would also be reduced to active Fe nanoclusters under the reductive gas from the reaction system to promote the growth of the NWs^39^. Meanwhile, alumina from the crucible could also be an effective mediator, playing an important role in controlling the concentration of reactants containing Si^16^. According to the results in the previous literature, alumina could react with silica to form mullite, which might be in a metastable phase when the temperature and the mole fractions of SiO_2_ and Al_2_O_3_ are within a certain range^16^,^41^,^42^,^43^. It is noteworthy that mullite could occur in the liquid phase separation process and generate liquid SiO_2_, which could react with other gases containing Si and then dissolve into the Fe nanoclusters to promote the growth of the Si_3_N_4_ NWs. Meanwhile, the liquid phase formation temperature of Al_2_O_3_-SiO_2_ would obviously be decreased if a small amount of oxide impurity was contained in the system of the Al_2_O_3_-SiO_2_. For example, the liquid phase formation temperature of the Al_2_O_3_-SiO_2_ system with a low content of alumina is 1595 °C, a value that decreases as low as 985 °C with the incorporation of 1% K_2_O^16^,^42^,^43^. It is noteworthy that there is approximately 4.5% Na_2_O in alumina, indicating that the liquid phase separation process would occur more easily, which would further promote the reaction between SiO_2_ and other gaseous products containing Si and would lead to continuous growth of the Si_3_N_4_ NWs. Therefore, alumina could play an important role in adjusting the content of reactive silicon to promote the growth of the Si_3_N_4_ NWs. Considering the role of alumina, an alumina-assisted VLS mechanism was proposed to explain the growth of the ultralong Si_3_N_4_ NWs. The possible growth process of the Si_3_N_4_ NWs is illustrated in Fig. 8 with three stages, including incubation, nucleation and growth, which could also be used to explain the larger yield and length of the products grown on the inner wall of the alumina crucible compared with the products obtained on the surface of powder mixture. Furthermore, elemental area scanning was carried out to study the composition of a single NW, as shown in Fig. 9, which demonstrated the presence of Al in the NW. This method also provides a simple procedure for preparing ultralong Si_3_N_4_ NWs doped with Al with special optical properties.

### Mechanical properties of Si_3_N_4_ NWs with different diameters

According to the results of the previous literature, the influence of diameter on the mechanical properties of NWs is very apparent^44^,^45^,^46^, while there are no reports on the effect of the diameters of Si_3_N_4_ NWs on their mechanical properties according to our survey. Therefore, this study attempted to quantitatively measure the Young’s moduli of Si_3_N_4_ NWs with different diameters by using Hertz’s model with *in situ* nanoindentation conducted in a hybrid SEM/SPM system. First, force-displacement curves were extracted to calculate the Young’s moduli of Si_3_N_4_ NWs with different diameters. It should be noted that the force-displacement curves obtained by the hybrid SEM/SPM system were the sum of the real penetration depth and the compressive deformation of the Si_3_N_4_ NWs under external force. To obtain the actual force-displacement curve, the single penetration depth (*h*) was extracted and the actual force-displacement curves were acquired after the single penetration depth (*h*) was obtained. Furthermore, the actual force-displacement curves were fitted with the general Sneddon’s expression (equation (1)), in which *F* was the applied force, *h* was the penetration depth, *n* was a spherical contact equal to 1.5, and *α* was an unknown constant, which was calculated from a power-law fit. Meanwhile, reference materials were utilized to estimate the tip radius using the following equation (2), where *α*_*ref*_ and *E*_*r(ref*)_ are the fitted parameter and reduced elastic modulus of the reference sample (a silicon wafer), respectively, and the reduced elastic modulus of the test sample *E*_*r*_ was calculated by equation (3).


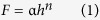



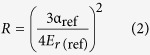



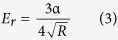


The Young’s modulus of sample *E*_*s*_ could be obtained using equation (4), where the subscripts *s* and *I* denote the sample and tip, respectively. *ν* is Poisson’s ratio, and *E* is Young’s modulus.


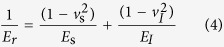


**Figure 10:**
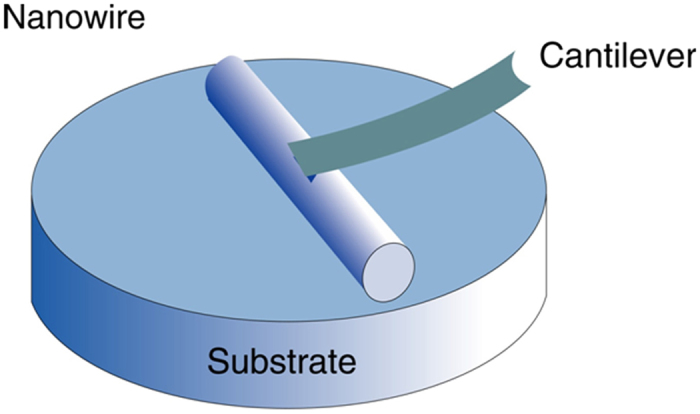
A schematic illustration for the model of nanoindentation in the system of SEM/SPM.

The calculation procedure described above could be used to calculate the Young’s moduli of Si_3_N_4_ NWs and a typical model of nanoindentation of NW is illustrated in Fig. 10 47,48.

**Figure 11:**
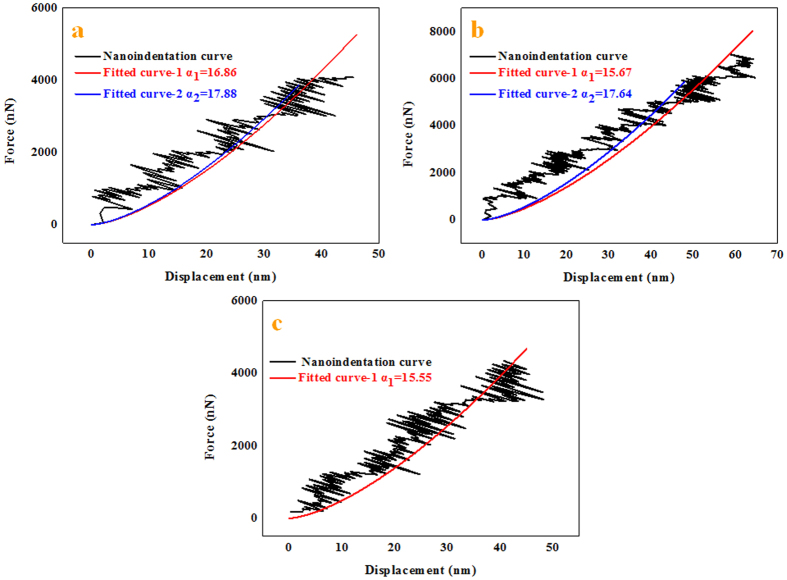
Force-displacement curves of different diameters of samples extracted from nanoindentation experiments with different fitted curves as displacement increased. The diameters were (**a**) 360 nm, (**b**) 480 nm and (**c**) 960 nm.

According to the obtained force-penetration depth curves, the Young’s moduli of Si_3_N_4_ NWs with different diameters were calculated by the above equations. Typical curves are shown in Fig. 11. To study the effect of diameter on the Young’s moduli of Si_3_N_4_ NWs, three different diameters of NWs were used, including approximately 360 nm, 480 nm and 960 nm. Loading curves were fit to the force-displacement curves to calculate the Young’s moduli of Si_3_N_4_ NWs. A general rule of the measurement of nanoindentation should be noted: the effective indentation depth should be deep enough to minimize the effect of surface, while the depth of indentation should also be less than 10% of the sample thickness when the sample is mounted on another substance, which can lead to differences the measured values due to the effect of the substrate^48^. Therefore, the effective displacements of penetration depth of the different diameters of Si_3_N_4_ NWs should be less than 36 nm, 48 nm and 96 nm, respectively. Meanwhile, owing to the limitation of the effective size of probe, the effective displacements of the penetration depth of the different diameters of Si_3_N_4_ NWs were selected to be 30 nm, 40 nm and 40 nm, respectively. From Fig. 11, it can be seen that the values of *α* calculated from a power-law fit changed with the displacement. The values of *α* decreased when the displacement increased thanks to the lower Young’s moduli of the silicon wafer compared with the Young’s moduli of the Si_3_N_4_ NWs. Furthermore, there was an obvious downward trend of the force-penetration depth curve, leading to a lower value of *α*, as shown in Fig. 11. According to the effective values of *α* (17.88, 17.64 and 15.55) of the different diameter Si_3_N_4_ NWs and the above equations, the Young’s moduli of the Si_3_N_4_ NWs were approximately 526.0 GPa, 498.2 GPa and 321.9 GPa, respectively, using the fitted parameter *α*_*ref*_ and the reduced elastic modulus *E*_*r(ref*)_ of the reference sample (silicon wafer) of 23.075 and 178.3 GPa, the properties of the silicon indenter tip (*ν*_*I*_ = 0.27 and *E*_*I*_ = 169 GPa) and the *ν*_*s*_ value for the Si_3_N_4_ NWs of 0.28^49^,^50^,^51^. The apparent Young’s moduli of the Si_3_N_4_ NWs decreased as the diameter of the NWs increased.

**Figure 12:**
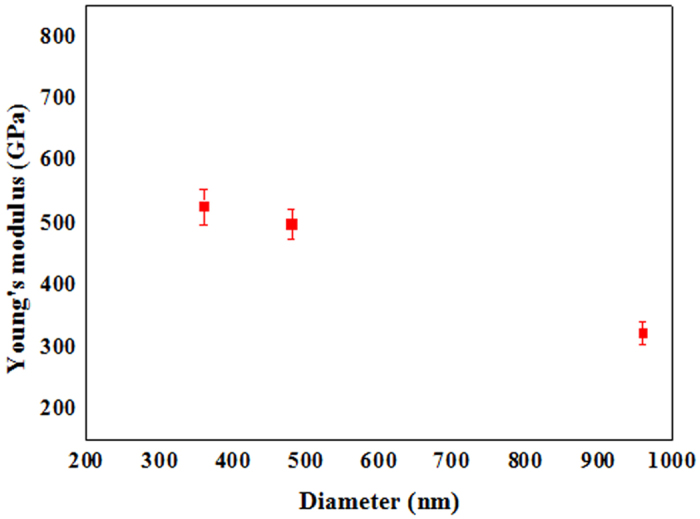
The value of Young’s modulus as a function of the diameter of the Si_3_N_4_ NWs.

The Young’s moduli of the Si_3_N_4_ NWs as a function of diameter are shown in Fig. 12. The date suggest that the Young’s moduli of the Si_3_N_4_ NWs decreased as the diameter of the NWs increased and that the calculated the Young’s modulus of a single Si_3_N_4_ NW with a 360 nm diameter close to its theoretical value, which was much greater than the Young’s modulus of the NWs with a diameter of 960 nm. Although the variations of the determined Young’s moduli may result from variable in the nanoindentation measurement, including tip radius, tip geometry, and different contact model, the main reason for this phenomenon could be ascribed to surface- and defect-free- related effects^52^. Generally, the effect of size on mechanical properties is obvious when the size is less than several tens of nanometers. For example, the Young’s moduli of ZnO NWs were found to decrease dramatically with increasing diameter, with the Young’s modulus reaching the value for bulk ZnO for diameters larger than 120 nm^53^, and Wu *et al*. observed that the Young’s moduli of Ag NWs with diameters fluctuating from 20 nm to 35 nm were scattered randomly in the range of 60–140 GPa^54^. However, the diameters of Si_3_N_4_ NWs were approximately 360 nm, 480 nm and 960 nm, which might not lead to the obvious downward trend observed for the Young’s moduli of the Si_3_N_4_ NWs as the diameter of the NWs increased from a general perspective. It is noteworthy that the effective displacements of the penetration depth of the tested NWs were only approximately 30 nm, 40 nm and 40 nm, respectively, and that the surface effects could not be ignored according to the previous literature, these effects could lead to a higher elastic modulus value^55^. Furthermore, there were no obvious defects exhibited in the Si_3_N_4_ NWs according to the above characterization of the NWs and this defect-free effect could also lead to a higher value of the mechanical properties similar to the reports about the measurement of elastic modulus^56^. According to the previous literature, the aspect ratio of length to diameter has played an important role in tests of NWs, which indicated that yield strength of specimens with an aspect ratio of 2:1 or higher in compression tests are approximately three times lower than in equivalent experiment using the same treatment conditions, as reported by D. Kiener *et al*.^57^,^58^. In addition, the lengths of Si_3_N_4_ NWs tested were approximately 20 μm for NWs with diameters of 360 nm, 80 μm for NWs with diameters of 480 nm, and 40 μm for NWs with diameters of 960 nm, giving aspect ratios of length to diameter of 55.6:1, 166.7:1and 41.7:1, respectively. Although the aspect ratios were different, the boundary and loading conditions in present study also were different to those used in the above literature report. Therefore, it is reasonable to believe that the diameters of the NWs could play an important role in determining the Young’s moduli of the Si_3_N_4_ NWs when compared to length and other factors, such as synthetic procedures, specimen geometry, and crystallographic orientation, as well as the nature of the experimental technique: uniform strain versus strain gradient-dominant and static versus dynamic deformation^52^, since the test process was so rapid and the different diameters of NWs were obtained in the same experiment. Meanwhile, the surface effects could also be caused by the diameters of the NWs compared to other differences in size such as length. Young’s modulus is expected to increase with decreasing crystal size because the probability of the existence of a defect would decrease as the size decreases more than the mean spacing between the defects in single-crystalline materials^52^. Furthermore, a similar trend could be found in the Young’s moduli of Si_3_N_4_ films as a function of thickness, as shown in Fig. S6, and the Young’s moduli of the Si_3_N_4_ films were found to decrease with the increase in the thickness of the films^51^,^59^,^60^, while the Young’s moduli approached a constant value when the thickness of the films was larger than 300 nm. Additional theoretical studies and further experiments are underway to understand the origin of size (including diameter, length, and others) dependence of the mechanical properties of Si_3_N_4_ NWs.

## Conclusions

Flow rate-induced diameter control in the synthesis of several millimeters-long Si_3_N_4_ NWs was achieved via a simple method. The average diameter of Si_3_N_4_ NWs increased when the flow rate of N_2_ gas increased, fluctuating from 290 nm from a flow rate of 100 ml/min to 425 nm from a flow rate of 400 ml/min. The diameter-dependence of the Young’s moduli of the Si_3_N_4_ NWs ranging from 360 nm to 960 nm in diameter was measured by *in situ* nanoindentation, which showed that the Young’s moduli of the Si_3_N_4_ NWs decreased as the diameter of the NWs increased from approximately 526.0 GPa to 321.9 GPa. VLS mechanisms were used to illustrate the growth models of Si_3_N_4_ NWs obtained from two different locations (i.e., the inner wall of the alumina crucible and the surface of the powder mixture), while evidence was found that alumina might be an effective mediator to promote the growth of NWs from the inner wall of the alumina crucible, which could be used to explain the larger yield and length of products growth on the inner wall of the alumina crucible compared with those of the products grown on the surface of the powder mixture. This method not only offers an effective means of preparing diameter-controlled ultralong Si_3_N_4_ NWs on an industrial scale but also provides significant guidance to those seeking to control the mechanical properties of the Si_3_N_4_ NWs, although additional theoretical studies and further experiments are required to understand the origin of the diameter dependence of the mechanical properties of the Si_3_N_4_ NWs.

## Methods

### Materials

Commercially available raw materials were used to prepare the ultralong Si_3_N_4_ nanowires described in this work. SiC powder (0.5 μm, Weifang Kaihua Micro-powder Co., Ltd., Shandong, China) and PCS (Mw = 2000, T_m_ = 180 °C, National University of Defense Technology, Changsha, China) were used as the raw materials.

### Preparation of ultralong Si_3_N_4_ NWs

First, 50 g SiC powder and 5 g PCS were combined as the powder mixture, which was ball-milled in tetrahydrofuran (THF) for 6 h with SiC balls and then dried in a rotary evaporator. A ceramic crucible was used to hold a certain amount of the powder mixture and then the crucible was placed into a tube corundum furnace. High-purity nitrogen gas was introduced into the furnace at a constant flow rate before heating and kept flowing during the whole experimental process. The furnace was heated from room temperature to 1400 °C with a heating rate of 3 °C/min, and this temperature was maintained for 2 h. The furnace was first cooled to 500 °C at the rate of 3 °C/min and then was naturally cooled to room temperature after heating was terminated. White wool-like products were formed on the inner wall of the alumina crucible and on the surface of the powder mixture.

### Characterization of ultralong Si_3_N_4_ NWs

Scanning electron microscopy (SEM, HELIOS NanoLab 600i, USA) equipped with energy dispersive spectroscopy (EDS), X-ray powder diffraction (XRD, X’PERT PRO MPD, Holland), Fourier transform infrared spectroscopy (FTIR, Spectrum Two, USA), transmission electron microscopy and high-resolution transmission electron microscopy (TEM and HRTEM, Tecnai G^2^-F30, USA) were employed to confirm the morphology, structure, and composition of the obtained products.

### *In situ* nanoindentation mechanical test

*In situ* nanoindentation experiments were conducted using a hybrid SEM/SPM system. The hybrid system relied on high-magnification SEM as a visual feedback system to indent the nanowires accurately with a cantilever probe. A more detailed description of the experimental setup can be found in the recent literature^47^.

## Additional Information

**How to cite this article:** Dong, S. *et al*. Facile synthesis of silicon nitride nanowires with flexible mechanical properties and with diameters controlled by flow rate. *Sci. Rep.* **7**, 45538; doi: 10.1038/srep45538 (2017).

**Publisher's note:** Springer Nature remains neutral with regard to jurisdictional claims in published maps and institutional affiliations.

## Supplementary Material

Supplementary Information
