# Supplementary material for: Facile synthesis of silicon nitride nanowires with flexible mechanical properties and with diameters controlled by flow rate
Source: Sci Rep. 2017 Mar 28;7:45538. doi: 10.1038/srep45538 (PMC5368666; doi:10.1038/srep45538)
Supplement: Supplementary Information [file srep45538-s1.pdf]

Supplementary Information

Facile synthesis of silicon nitride nanowires with flexible mechanical properties and with diameters controlled by flow rate

Shun Dong, Ping Hu*, Xinghong Zhang*, Yuan Cheng, Cheng Fang, Jianguo Xu, Guiqing Chen

Science and Technology on Advanced Composites in Special Environment Laboratory, Harbin Institute of Technology, Harbin 150001, PR China

****Corresponding author*: Tel/fax: +86 451 86403016.**

E-mail address: Hupinghit88@163.com (P. Hu); zhangxh@hit.edu.cn (X.H. Zhang)

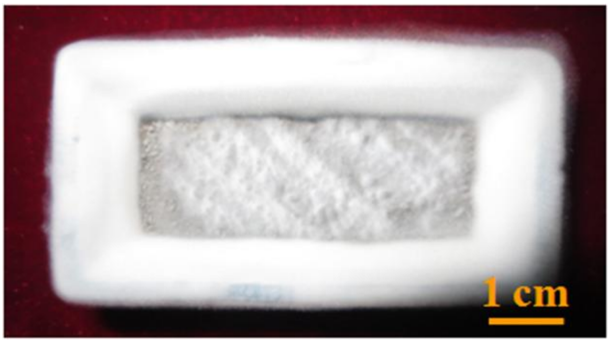

Fig. S1 The macroscopic morphology of the achieved products synthesized with 100 ml/min of N_2 at 1400 ^oC.

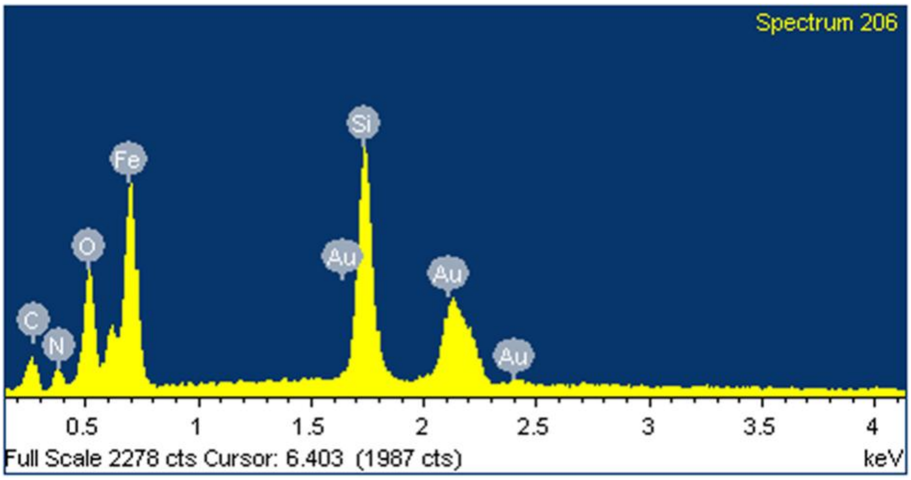

Fig. S2 A typical EDS spectrum of the tip of a single Si_3N_4 NW grown on the inner wall of the alumina crucible.

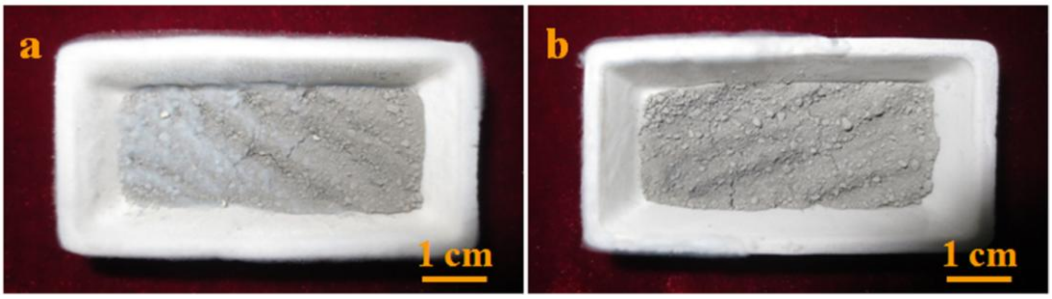

Fig. S3 The macroscopic morphology of as-prepared products synthesized with different flow rates of N_2 at 1400 ^oC. The flow rates were (a) 200 ml/min and (b) 400 ml/min.

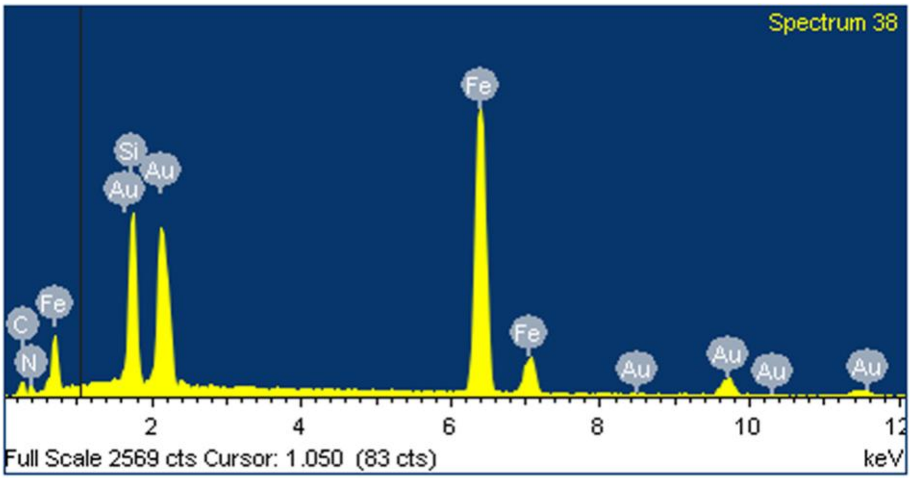

Fig. S4 A typical EDS spectrum of the tip of a single Si_3N_4 NW grown on the surface of the powder mixture.

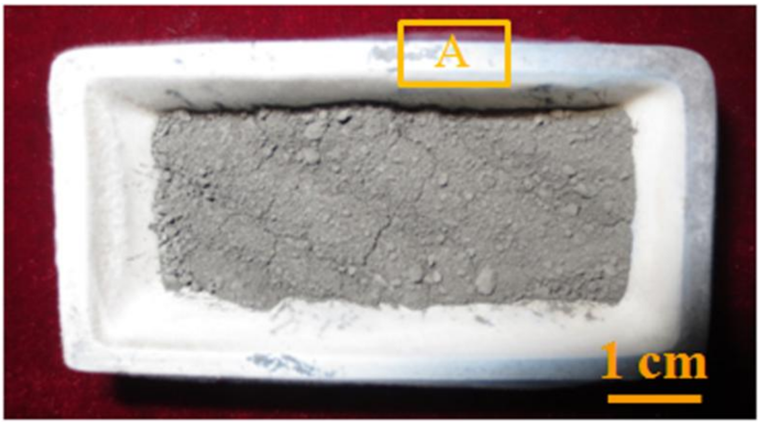

Fig. S5 The macroscopic morphology of the products synthesized with 100 ml/min of N_2 at 1400 ^oC using the pretreated powder, in which SiC was treated in HCl for 60 h to remove Fe and then ball-milled with 10 wt% PCS.

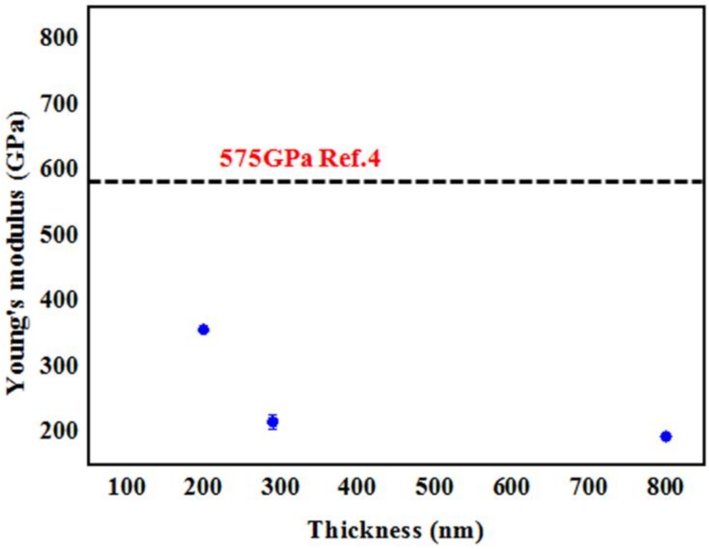

Fig. S6 The value of Young's modulus as a function of the thickness of Si_3N_4 films reported in the literatures[1-4].

References

1 Brantley, W. A. Calculated elastic constants for stress problems associated with semiconductor devices. *J. Appl. Phys.* **44**, 534-535 (1973).

2 Oliver, W. C., & Pharr, G. M. Measurement of hardness and elastic modulus by instrumented indentation: Advances in understanding and refinements to methodology. *J. Mater. Res.* **19**, 3-20 (2004).

3 Tabata, O., Sugiyama, S., & Takigawa, M. Control of internal stress and Young's modulus of Si_3N_4 and polycrystalline silicon thin films using the ion implantation technique. *Appl. Phys. Lett.* **56**, 1314-1316 (1990).

4 Jing, G.Y., Ji, H., Yang, W.Y., Xu, J., Yu, D.P., Study of the bending modulus of individual silicon nitride nanobelts via atomic force microscopy. *Appl. Phys. A*, **82**, 475-478 (2006).
